# Defects in Actin Dynamics Lead to an Autoinflammatory Condition through the Upregulation of CXCL5

**DOI:** 10.1371/journal.pone.0002701

**Published:** 2008-07-16

**Authors:** Angela M. Verdoni, Richard S. Smith, Akihiro Ikeda, Sakae Ikeda

**Affiliations:** 1 Department of Medical Genetics, University of Wisconsin-Madison, Madison, Wisconsin, United States of America; 2 The Jackson Laboratory, Bar Harbor, Maine, United States of America; University of Birmingham, United Kingdom

## Abstract

**Background:**

Destrin (DSTN) is a member of the ADF/cofilin family of proteins and is an important regulator of actin dynamics. The primary function of destrin is to depolymerize filamentous actin into its monomeric form and promote filament severing. While progress has been made in understanding the biochemical functions of the ADF/cofilin proteins, the study of an animal model for cells deficient for DSTN provides an opportunity to investigate the physiological processes regulated by proper actin dynamics *in vivo*. A spontaneous mouse mutant, corneal disease 1(*corn1*), is deficient for DSTN, which causes epithelial hyperproliferation and neovascularization in the cornea. *Dstn^corn1^* mice exhibit an actin dynamics defect in the cornea as evidenced by the formation of actin stress fibers in the epithelial cells. Previously, we observed a significant infiltration of leukocytes into the cornea of *Dstn^corn1^* mice as well as the upregulation of proinflammatory molecules. In this study, we sought to characterize this inflammatory condition and explore the physiological mechanism through which a loss of *Dstn* function leads to inflammation.

**Methodology/Principal Findings:**

Through immunofluorescent analyses, we observed a significant recruitment of neutrophils and macrophages to the *Dstn^corn1^* cornea, demonstrating that the innate immune system is spontaneously activated in this mutant. The inflammatory chemokine, CXCL5, was ectopically expressed in the corneal epithelial cells of *Dstn^corn1^* mice, and targeting of the receptor for this chemokine inhibited neutrophil recruitment. An inflammatory reaction was not observed in the cornea of allelic mutant strain, *Dstn^corn1-2J^*, which has a milder defect in actin dynamics in the corneal epithelial cells.

**Conclusions/Significance:**

This study shows that severe defects in actin dynamics lead to an autoinflammatory condition that is mediated by the expression of CXC chemokines.

## Introduction

Assembly and disassembly of filamentous actin (F-actin) within a eukaryotic cell, which are referred to as “actin dynamics”, play an essential role in a number of biological processes such as cell motility, cell polarization, contractile force generation, cell division and membrane dynamics [1]–[3]. Actin dynamics are spatially and temporally regulated through the function of actin-binding proteins [1], including those that belong to the actin depolymerizing factor (ADF)/cofilin family. This family of proteins influence actin filament turnover by binding to actin subunits in F-actin [4], [5], which results in filament severing and the enhancement of subunit dissociation [6], [7]. Through these functions, the ADF/cofilin family regulates the balance between intracellular F- and G-actin pools. Unicellular organisms have only one gene encoding an ADF/cofilin protein, whereas multicellular organisms have several isoforms [8]. Mammals produce three ADF/cofilin family members with different expression patterns and biochemical properties: destrin (DSTN; also known as actin depolymerizing factor or ADF) (MGD ID MGI:1929270, Entrez Protein ID NP_062745) cofilin 1 (CFL1) (MGD ID MGI:101757, Entrez Protein ID AAH94357) and cofilin 2 (CFL2) (MGD ID MGI:101763, Entrez Protein ID AAH07138) [8]. CFL1 is ubiquitously expressed in most cell types throughout development and adulthood, while CFL2 is a muscle specific isoform with the weakest depolymerization activity [8]. DSTN shows the strongest depolymerization activity out of all family members and is expressed in epithelial and endothelial cells of multiple tissues [8], [9].

Studies using model organisms with perturbed ADF/cofilin functions have revealed cellular mechanisms in which ADF/cofilins have significant roles, and also the pathological consequences of their loss. ADF/cofilin deficiencies are lethal in yeast and *Drosophila* [10]–[12]. In *C. elegans*, the *unc-60* gene encodes two homologous ADF/cofilin proteins, UNC-60A and UNC-60B, through alternative splicing. Mutations in *unc-60* that affect the B isoform result in paralysis and those that affect both isoforms result in lethality [2], [13], [14]. This suggests a role for these proteins in the development of muscle and other tissues. Complete loss of CFL1 in mice results in embryonic lethality with defective neural crest cell migration and a lack of neural tube closure [15]. Neuronal cell-specific targeting of CFL1 further revealed its function in neuronal migration and cell cycle control in the cerebral cortex [16]. Mice homozygous for spontaneous mutations in the *Dstn* gene have been identified. Mice homozygous for a null mutation in *Dstn* (*Dstn^corn1^*) [17] develop a thickened corneal epithelium due to epithelial cell hyperproliferation, which is followed by stromal neovascularization [18]. Mice homozygous for a missense mutation in *Dstn* (*Dstn^corn1-2J^*) display milder corneal phenotypes. The thickening of the corneal epithelium is less severe, and stromal neovascularization does not occur in this mutant [17]. These phenotypic differences could be due to the different level of gene expression changes that the allelic *Dstn* mutations lead to [19]. Mutant phenotypes appear to be restricted to the cornea, where the main ADF/cofilin family member expressed is DSTN [17]. In other tissues, the loss of DSTN is likely compensated by CFLs. Based on these notions, the cornea of *Dstn* mutant mice represent a unique model in which the *in vivo* function of DSTN can be studied. Consistent with a loss of normal DSTN function, F-actin accumulation was observed in the corneal epithelium of both *Dstn^corn1^* and *Dstn^corn1-2J^* mice, although at a much greater level in *Dstn^corn1^* mice [17], [19]. A biochemical analysis also confirmed that actin dynamics are perturbed in *Dstn* mutants [19]. Within the cornea, DSTN is mainly expressed in the epithelial layers [17], [20], suggesting that actin dynamics defects in the corneal epithelial cells result in the pathological changes observed in these mice.

In addition to the originally described phenotypes, we observed infiltration of leukocytes into the cornea of *Dstn^corn1^* mice [20]. At 4 weeks of age, there were significantly more CD45 positive leukocytes in *Dstn^corn1^* mice compared with wild-type controls [20]. This spontaneous recruitment of inflammatory cells in the *Dstn^corn1^* cornea represents another physiological/pathological consequence of actin dynamics defects *in vivo*. In this study, we sought to characterize this inflammatory condition and explore the underlying molecular mechanism through which the *Dstn* mutation leads to inflammation. We show that neutrophils and macrophages are selectively recruited to the *Dstn^corn1^* cornea and that neutrophil infiltration is mediated by the expression of CXC chemokines. Inflammation was not observed in the cornea of allelic mutant strain, *Dstn^corn1-2J^*, which shows milder actin dynamics defects [17], [19]. These results demonstrate that the *Dstn^corn1^* mutation, which causes severe defects in actin dynamics in the corneal epithelial cells [17], [19], lead to an autoinflammatory condition in the cornea that is mediated by the expression of CXC chemokines.

## Results

### Neutrophils and macrophages are recruited to the corneal stroma of *Dstn^corn1^* mice

**Figure 1:**
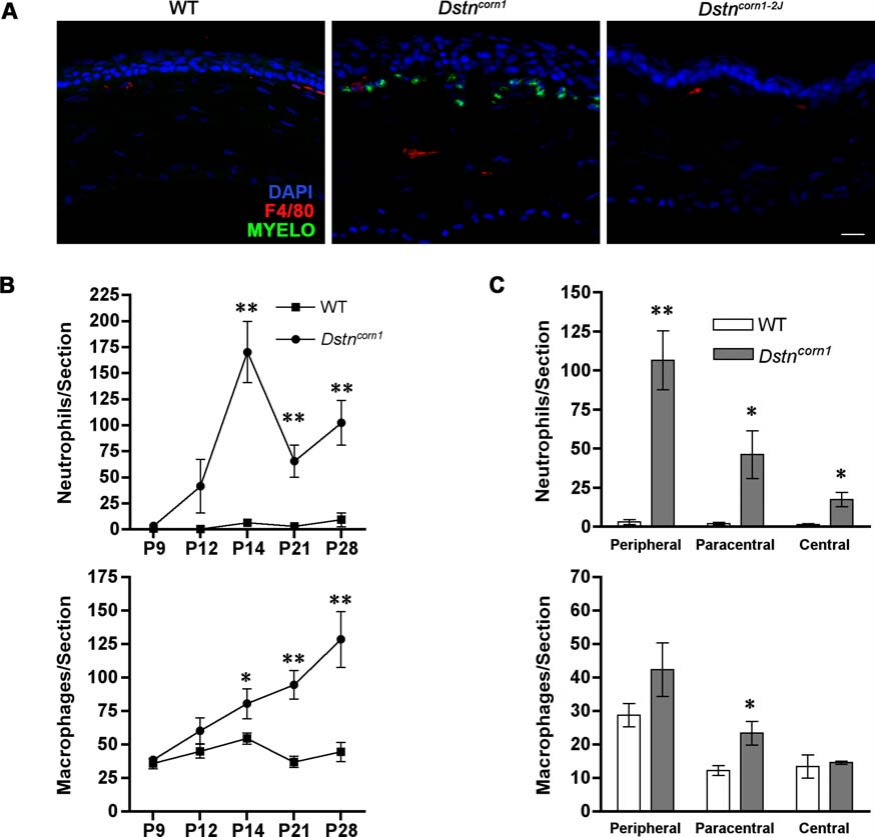
Inflammatory cell recruitment in the corneas of *Dstn* mutant mice. (A): Immunofluorescent staining for macrophages (F4/80, red) and neutrophils (myeloperoxidase, green) in WT, *Dstn^corn1^*, and *Dstn^corn1-2J^* cornea at P14. Neutrophils are recruited to the stromal area in *Dstn^corn1^*, but not WT or *Dstn^corn1-2J^* cornea. Sections are counterstained with DAPI (blue). The central region of the cornea is shown in all images. The scale bar corresponds to 10 µm. (B): Quantification of inflammatory cells in WT and *Dstn^corn1^* mice. Accumulation of neutrophils is observed in *Dstn^corn1^* cornea as compared to WT beginning at P14. Significantly higher numbers of macrophages are also present in *Dstn^corn1^* cornea beginning at P14, and most significantly at P28. Error bars represent standard error of measurement (SEM). * denotes statistical significance by *t*-test. (C): Quantification of inflammatory cells in different regions of the cornea shows that the greatest numbers of neutrophils and macrophages accumulate in the peripheral region, the area adjacent to the limbal vasculature. Error bars represent SEM. * denotes statistical significance by *t*-test.

Previously, we observed spontaneous recruitment of CD45 positive leukocytes into the cornea of *Dstn^corn1^* mice [20]. To determine which cell types are infiltrating the cornea, we performed immunofluorescent analyses using myeloperoxidase as a marker for neutrophils [21], [22], F4/80 for macrophages [23], CD3 for T cells [24] and CD45R/B220 for B cells [25], [26]. We also performed these analyses on the cornea of *Dstn^corn1-2J^* mice to determine if an inflammatory response is occurring in this *Dstn* mutant. Neutrophil infiltration was observed only in the cornea of *Dstn^corn1^* mice and not in wild-type or *Dstn^corn1-2J^* mice at P14 (Fig 1A). A quantitative analysis at various timepoints revealed that, while very few neutrophils were observed in the cornea of A.BY wild-type mice at any timepoint, infiltration of neutrophils to the *Dstn^corn1^* cornea is evident by P12. The number of neutrophils is significantly higher in the cornea of *Dstn^corn1^* mice beginning at P14 and persisting through P28 (Fig 1B). The number of macrophages in the cornea of *Dstn^corn1^* mice was also significantly increased compared to that of A.BY wild-type mice at P14, P21 and P28 (Fig 1A,B). Neutrophils and macrophages were most abundant in the peripheral region of the *Dstn^corn1^* cornea at P14 (Fig 1C). For comparison, we quantified the number of neutrophils and macrophages in the cornea of *Dstn^corn1-2J^* mice at P14 and P21. The number of neutrophils and macrophages were not significantly increased in the cornea of *Dstn^corn1-2J^* mice compared with B6 wild-type mice at P14 or P21 (data not shown). A significant increase in the number of CD3-positive T cells and CD45R/B220-positive B cells was not observed in the cornea of *Dstn^corn1^* or *Dstn^corn1-2J^* mice at P14 as compared to controls (data not shown). This demonstrates that an inflammatory response resulting in the specific recruitment of neutrophils and macrophages occurs only in the *Dstn^corn1^* cornea.

**Figure 2:**
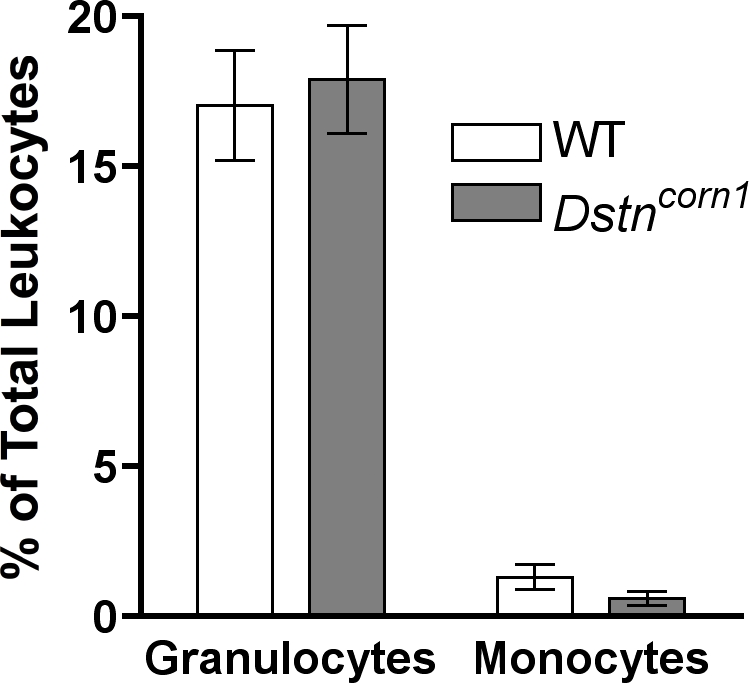
Assessment of granulocyte and monocyte ratios in the peripheral leukocyte population of A.BY WT and *Dstn^corn1^* mice. A flow cytometric analysis revealed that the ratios of granulocytes, which are comprised mostly of neutrophils, and monocytes, which are the precursor to macrophages, are not significantly different between A.BY WT and *Dstn^corn1^* mice. Ratios are expressed as the percentage of cells compared to the total leukocyte number. Error bars represent SEM.

To determine if the infiltration of neutrophils and macrophages to the *Dstn^corn1^* cornea is the result of a systemic change in the leukocyte population, we examined the relative numbers of granulocytes and monocytes in the peripheral blood of *Dstn^corn1^* mice. We isolated blood from adult A.BY WT and *Dstn^corn1^* mice. A flow cytometric analysis of the white blood cells demonstrated that the overall leukocyte percentage of granulocytes (17.06±1.83 versus 17.92±1.81), which are comprised mostly of neutrophils [27], [28], and monocytes (1.33±.406 versus .617±.243), which are the precursors to the macrophage cell type, were not significantly different between A.BY WT and *Dstn^corn1^* mice, respectively (Fig 2). A similar result was obtained upon quantification of the neutrophil and monocyte cell types in peripheral blood smears stained with Diff-Quik, which facilitates the morphological identification of leukocyte subtypes. No significant difference for the neutrophil and monocyte population was observed between A.BY WT and *Dstn^corn1^* mice (data not shown). This demonstrated that the infiltration of inflammatory cells in the *Dstn^corn1^* cornea is likely due to local signals in the cornea rather than a systemic change in the numbers of leukocytes.

### Ectopic expression of CXCL5 in the corneal epithelium of *Dstn^corn1^* mice

**Figure 3:**
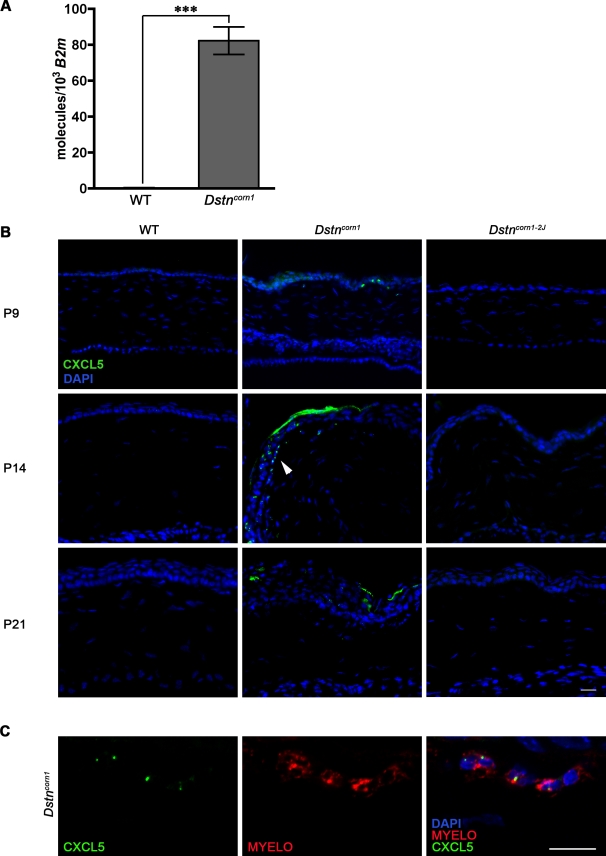
Expression of CXCL5 in *Dstn* mutant mice. (A): Real-time PCR analysis for *Cxcl5*. The relative expression level of *Cxcl5* is expressed as the number molecules per 1000 *B2m* molecules, and is significantly higher in the *Dstn^corn1^* cornea as compared to WT. Error bars represent SEM. * denotes statistical significance by *t*-test. (B): Immunofluorescent staining for CXCL5 in *Dstn* mutant mice. Positive immunoreactivity for CXCL5 (green) in the corneal epithelium of *Dstn^corn1^* mice at P9, P14, and P21 and in some stromal cells (arrowhead). CXCL5 is not expressed in WT or *Dstn^corn1-2J^* epithelium at any time points tested. The central region of the cornea is shown in all P9 and P21 images. The peripheral region is shown in all P14 images. (C): Immunofluorescent staining for CXCL5 (green) with the intracellular neutrophil marker, Myeloperoxidase (red). Single slice confocal images demonstrate that CXCL5 is localized to the cytoplasm of neutrophils invading the *Dstn^corn1^* cornea. Sections are counterstained with the nuclear marker, DAPI (blue). The central region of the cornea is shown. All scale bars correspond to 10 µm.

In our previous microarray analysis comparing the gene expression profiles of the *Dstn^corn1^*, *Dstn^corn1-2J^* and wild-type cornea, we observed that inflammation associated gene ontology (GO) terms were over-represented in the list of genes upregulated in the *Dstn^corn1^* cornea compared to the wild-type cornea ([19]; the microarray data set is available from NCBI Gene Expression Omnibus (http://www.ncbi.nlm.nih.gov/geo/) with GEO Accession number GSE9743 (the accession link for reviewers: http://www.ncbi.nlm.nih.gov/geo/query/acc.cgitokenjfopnskcesamipsaccGSE9743). These terms were not over-represented in the list of upregulated genes for the *Dstn^corn1-2J^* cornea. One of such inflammation-related molecules that are upregulated in the *Dstn^corn1^* but not in the *Dstn^corn1-2J^* cornea, chemokine (C-X-C motif) ligand 5 (CXCL5) (MGD ID MGI:1096868, Entrez Protein ID NP_033167) is a potent neutrophil chemoattractant [29], [30] that was originally isolated from epithelial cells and fibroblasts [31]. These characteristics of CXCL5 make it a candidate molecule that possibly participates in the induction of the inflammatory condition observed in the *Dstn^corn1^* cornea. By quantitative real-time PCR (qPCR) analysis, we confirmed that the expression of *Cxcl5* is greatly upregulated in the cornea of *Dstn^corn1^* mice compared to that in wild-type mice (Fig 3A; p = 0.0004). We then tested the temporal and spatial expression pattern of the CXCL5 protein by immunofluorescence. This analysis demonstrated that CXCL5 is expressed in the corneal epithelial cells of *Dstn^corn1^* mice as early as P9 (in one of three eyes tested), and in all *Dstn^corn1^* samples tested at P14 and P21 (n = 3 per timepoint) (Fig 3B). CXCL5 expression was observed mostly in the superficial layers of the thickened *Dstn^corn1^* epithelium (P14 and P21). Additionally, we detected the localization of CXCL5 to some cells in the corneal stroma of *Dstn^corn1^* mice (Fig 3B, arrowhead). To determine which cell type in the stroma CXCL5 is localized to, we first performed double labeling with an antibody detecting the intracellular neutrophil specific protein, myeloperoxidase, [21], [22]. This experiment revealed that CXCL5 localization in the corneal stroma is restricted to invading neutrophils. A single slice confocal image demonstrated that CXCL5 signal is observable within the cytoplasm (Fig 3C). Neutrophils have been shown to express CXCL5 and it functions as a chemoattractant through the neutrophil cell surface receptor, IL8RB [32] (Reviewed in [29]). Thus, the localization of CXCL5 within the cells may represent the production of CXCL5 by neutrophils or the binding and internalization of CXCL5 bound to IL8RB, a cell surface receptor internalized upon chemokine stimulation 33–35. CXCL5 is not expressed in the corneas of either wild-type or *Dstn^corn1-2J^* mice.

### Targeting of the receptor for CXC chemokines upregulated in *Dstn^corn1^* cornea inhibits neutrophil recruitment

**Figure 4:**
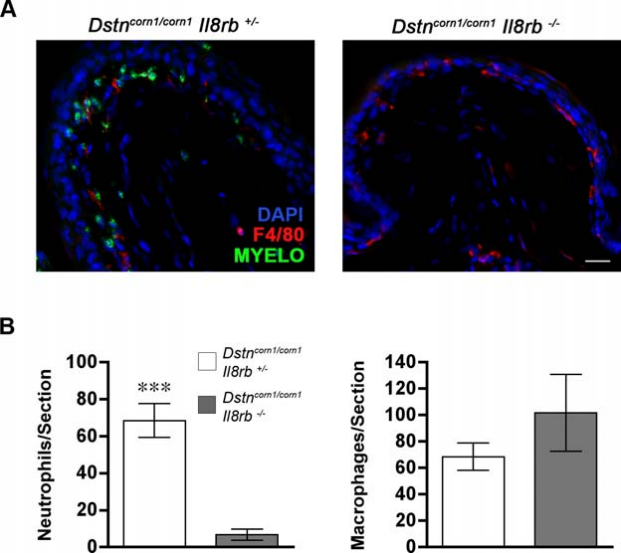
Inflammatory cell recruitment in the compound mutants for *Dstn* and *Il8rb*. (A): Representative images showing a lack of myeloperoxidase-positive neutrophils (green) in the cornea of *Dstn^corn1/corn1^ Il8rb^−/−^* mice at P21. The peripheral region of the cornea is shown. The scale bar corresponds to 10 µm. (B): Quantification of neutrophils and macrophages in *Dstn^corn1/corn1^ Il8rb^+/−^* and *Dstn^corn1/corn1^ Il8rb^−/−^* cornea. Neutrophil recruitment is almost completely inhibited in *Dstn^corn1/corn1^ Il8rb^−/−^* cornea. A tendency for greater macrophage recruitment, although not statistically significant, is observed. Error bars represent SEM. * denotes statistical significance by *t*-test.

CXCL5 is one of the known ligands of the interleukin 8 receptor beta (IL8RB) (MGD ID MGI:105303, Entrez Protein ID NP_034039) that have chemotactic properties [36]. In addition to CXCL5, our previous microarray analysis identified 3 other CXC chemokines that are ligands of IL8RB as upregulated in the *Dstn^corn1^* cornea [19]. To determine if a pathway initiated by these ligands through their receptor mediates the inflammatory cell infiltration in the *Dstn^corn1^* cornea, we generated *Dstn^corn1^* mice that are also deficient for IL8RB. We examined the recruitment of neutrophils and macrophages to the cornea of these mice, and determined that neutrophil recruitment is almost completely inhibited in *Dstn^corn1/corn1^ Il8rb^−/−^* cornea as compared to *Dstn^corn1/corn1^ Il8rb^+/−^* cornea at P21 (6.8±3.0 versus 68.59±9.1, p = 0.0002) (Fig 4A,B). Even though there tended to be a greater number of macrophages in *Dstn^corn1/corn1^ Il8rb^−/−^* cornea as compared to *Dstn^corn1/corn1^ Il8rb^+/−^* cornea, this difference did not reach statistical significance (101.9±29.13 versus 68.75±10.48, p = 0.345) (Fig 4A,B).

### Neovascularization in *Dstn^corn1^* mice is not affected by the absence of neutrophils

**Figure 5:**
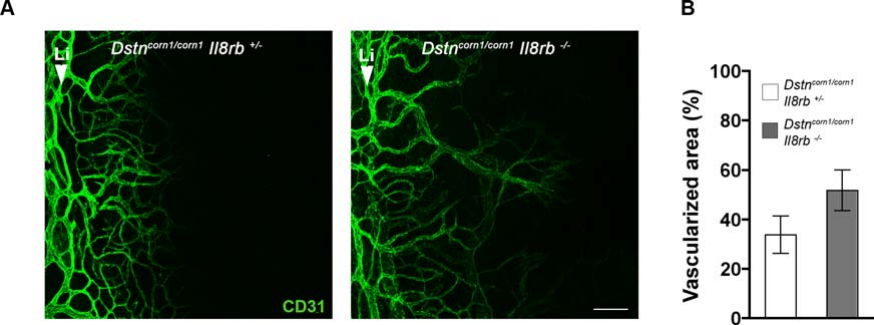
Neovascularization in the cornea of compound mutants for *Dstn* and *Il8rb*. (A): Representative images showing CD31^+^ (green) blood vessel growth from the limbus (Li) in P21 *Dstn^corn1/corn1^ Il8rb^+/−^* and *Dstn^corn1/corn1^ Il8rb^−/−^* cornea. The scale bar corresponds to 100 µm. (B): Quantification of vascularized area at P21 in whole mount cornea. A tendency for a greater vascularized area is observed in *Dstn^corn1/corn1^ Il8rb^−/−^* cornea, although not statistically significant as determined by Mann-Whitney test. Error bars represent SEM.

Since neutrophils have been shown to have angiogenic properties [37], we tested whether the absence of neutrophil recruitment as a result of the *Il8rb* deficiency also affects neovascularization in *Dstn^corn1^* mice. The vessel growth in the cornea of *Dstn^corn1/corn1^ Il8rb^+/−^* and *Dstn^corn1/corn1^ Il8rb^−/−^* mice was compared by measuring the corneal area that contains CD31-positive blood vessels in whole mount cornea. We did not observe a significant difference in the percentages of vascularized area between *Dstn^corn1/corn1^ Il8rb^+/−^* and *Dstn^corn1/corn1^ Il8rb^−/−^* cornea (33.99±7.51 versus 51.94±8.252, p = 0.197) (Fig 5A,B).

## Discussion

In this study, we extended our previous finding and characterized the spontaneous inflammatory condition that occurs as a result of the *Dstn^corn1^* mutation. We determined that increased numbers of neutrophils and macrophages, but not T or B cells, exist in the cornea of *Dstn^corn1^* mice compared to wild-type mice. These results demonstrate that the innate immune system is spontaneously activated in this mutant. This condition is reminiscent of what is observed in autoinflammatory diseases. The concept of autoinflammatory diseases has been recently introduced as disorders characterized by seemingly unprovoked inflammation [38]. They include hereditary periodic fever syndromes, granulomatous inflammation, familial urticarial syndromes, complement disorder and vasculitis syndrome [38]. These conditions are primarily caused by dysregulation of the innate immune system without the primary involvement of the adaptive immune system. Identification of the responsible genes for genetic autoinflammatory diseases have revealed that disturbances in pathways associated with innate immune cell function (reviewed in [39]), including abnormal signaling in cytokine pathways such as TNF (MGD ID MGI:104798, Entrez Protein ID AAC82484) and interleukin-1 beta (MGD ID MGI:96543, Entrez Protein ID AAH11437) [40], and mutations in proteins associated with bacterial sensing [38], [41] are involved in the disease causing mechanism. Our findings in the *Dstn^corn1^* mice demonstrate that mutations in an actin depolymerizing molecule and resulting actin dynamics defects could lead to an autoinflammatory condition. Even though the disease appearances and affected tissues are different, it is possible that similar molecular defects and pathways are involved in the induction of autoinflammatory diseases.

In this study, we sought to reveal the molecular pathway through which the *Dstn^corn1^* mutation leads to the autoinflammatory condition. Based on our observation that CXC chemokines are upregulated in *Dstn^corn1^* mice (Fig 3; [19]), we examined whether these molecules mediate the recruitment of inflammatory cells to the cornea in this model. By combining the null mutation of the receptor for these chemokines (*Il8rb*) with the *Dstn^corn1^* mutation, our study showed that neutrophils are not recruited to the cornea of *Dstn^corn1^* mice in the absence of *Il8rb*. Taken together, these results suggest that the upregulation of CXC chemokines is primarily responsible for the recruitment of neutrophils to the cornea of *Dstn^corn1^* mice. In contrast, the number of macrophages was not significantly different in the compound mutant mice. Thus, the expression of CXC chemokines is responsible for the selective recruitment of neutrophils to the cornea of *Dstn^corn1^* mice. IL8RB dependent recruitment of neutrophils is observed in acute inflammation models [42] [43] [44], but not in chronic inflammation models [45], [46]. Since neutrophil recruitment to the cornea of *Dstn^corn1^* mice is almost completely inhibited when mice are deficient for *Il8rb*, the mechanism causing inflammation in *Dstn^corn1^* mice may be similar to those in acute inflammatory models. However, the *Dstn^corn1^* mouse is a unique model since an inflammatory condition is not induced by an infection or other experimental manipulations, but rather is the result of a mutation in the endogenous *Dstn* gene.

As observed for CXCL5 in this study, differential expression of many molecules occur in the cornea of *Dstn^corn1^* mice compared to wild-type mice. Gene expression profiling in our previous study revealed dramatic alteration of gene expression in the *Dstn^corn1^* cornea, with 1,226 annotated genes differentially expressed [19]. We have examined the spatial expression pattern of some of these molecules, including CXCL5 and actin related molecules, and found that the expression change occurs mainly in the corneal epithelial cells (Fig 3; [19]). This is consistent with the fact that *Dstn* is primarily expressed in the epithelium within the cornea [17], [20]. F-actin accumulation was also observed in the corneal epithelial cells of *Dstn* mutants [17], [19], demonstrating that actin dynamics are affected by *Dstn* mutations in these cells. Based on these findings, we hypothesize that there are molecular pathways affected by the *Dstn* mutations and actin dynamics defects that lead to alteration of gene expression in corneal epithelial cells. Further study should reveal the link between actin dynamics and the expression of these genes.

We observed that neutrophil infiltration into the cornea of *Dstn^corn1^* mice is evident by 12 days of age, which is before the onset of neovascularization at P14. Neutrophils have been shown to express angiogenic factors [47] [48] and to promote neovascularization in several experimental models [37], [49], [50]. Therefore, we speculated that neutrophils might play a role in the induction of neovascularization in this mouse model. However, compound mutants for *Dstn* and *Il8rb* exhibit neovascularization comparable to that observed in *Dstn^corn1^* mice. These results suggest that neutrophil recruitment and the expression of IL8RB ligands, including CXCL5, are not the cause of neovascularization in *Dstn^corn1^* mice, but may be changes that occur in parallel with angiogenesis. Since macrophages have been also shown to express angiogenic factors [51]–[54] and to modulate angiogenesis in some animal models [55]–[60], macrophage activation and/or recruitment could be another candidate as the source responsible for abnormal angiogenesis in *Dstn^corn1^* mice.

An autoinflammatory condition was not observed in the allelic mutant strain, *Dstn^corn1-2J^*. In agreement with this finding, inflammation related GO terms were not identified in the list of differentially expressed genes for this mutant [19]. Also, the expression of the pro-inflammatory molecule, CXCL5, in the corneal epithelial cells was detected only in *Dstn^corn1^* and not in *Dstn^corn1-2J^* mice. As previously described, the *Dstn^corn1^* mutation is the deletion of the gene (null mutation of the *Dstn* gene), whereas the *Dstn^corn1-2J^* mutation is a point mutation, which may only affect some part of DSTN function and result in a reduction of DSTN activity [17]. The *Dstn^corn1^* mutation results in a much more pronounced F-actin accumulation compared to the *Dstn^corn1-2J^* mutation [17], [19]. The phenotypic difference between the allelic mutants, including the presence and absence of an inflammatory condition, might be associated with the level of remaining DSTN activity in the epithelial cells. The molecular pathway leading to the autoinflammatory condition in *Dstn^corn1^* mice may not be affected by the milder actin dynamics defects caused by the *Dstn^corn1-2J^* mutation. Consistent with this notion, CXCL5 expression was observed primarily in the superficial layers in thickened *Dstn^corn1^* epithelium, where F-actin accumulation is most severe [19].

In conclusion, the *in vivo* loss of an actin depolymerizing molecule, DSTN, leads to an autoinflammatory condition in the cornea. The defects in actin dynamics observed in *Dstn^corn1^* mice likely lead to a change in gene expression in the corneal epithelial cells [19], which results in the expression of proinflammatory molecules. This change in gene expression then leads to the specific recruitment of inflammatory cells, in particular neutrophils and macrophages, to the cornea. Further analysis of this mouse model should lead to the identification of the link between actin dynamics and the molecular mechanism underlying aberrant inflammatory gene expression.

## Materials and Methods

### Mice

A.BY *H2^bc^ H2-T18^f^*/SnJ (A.BY wild-type) (Stock # 000140), A.BY *H2^bc^ H2-T18^f^*/SnJ-*Dstn^corn1^*/J (*Dstn^corn1^*) (Stock # 001649), C57BL/6J (B6 wild-type) (Stock # 000664), C57BL/6JSmn-*Dstn^corn1-2J^*/J (*Dstn^corn1-2J^*) (Stock # 002623), and C.129S2(B6)-*Il8rb^tm1Mwm^*/J (*Il8rb^−/−^*) (Stock # 002724) mice were obtained from The Jackson Laboratory (Bar Harbor, ME). All mouse procedures were performed in accordance with the protocols approved by the Animal Care and Use Committee at the University of Wisconsin-Madison, and conform to the ARVO statement for the use of animals in Ophthalmic and Vision Research.

### Genotyping

All genotyping was carried out by polymerase chain reaction (PCR). For destrin genotyping, PCR primers, mADF-F31 (5′ GTCCCATGAATGTGAATTGC 3′) and mADF-R28 (5′ CCCTGGTGACCTTTCCTTATC 3′), were used for amplification of the wild type (WT) allele. Primers, mADF-F32 (5′ GCCACATCATTAGCTTTTGAAG 3′) and mADF-R30 (5′ TGGCACTCCTGCTGTCAC 3′), were used for amplification of the *Dstn^corn1^* allele. Genotyping for the interleukin 8 receptor beta (*Il8rb*) was performed as described previously [61].

### Real-time PCR analysis

Total RNA was isolated from corneas of A.BY wild-type and *Dstn^corn1^* mice using Trizol reagent (Invitrogen) and treated with DNaseI (Ambion). cDNA was synthesized from 2 µg of total RNA using Retroscript cDNA synthesis kit (Ambion). PCR reactions for chemokine (C-X-C motif) ligand 5 (*Cxcl5*) were performed in triplicate along with triplicate reactions of an internal control gene, beta-2 microglobulin (*B2m*), using SYBR Green PCR Master Mix (Applied Biosystems). Gene specific primers were designed to amplify 180–200 bp products: *Cxcl5* (forward) CGTAACTCCAAAAATTAATCCCAAA, (reverse) CGAGTGCATTCCGCTTAGCT; *B2m* (forward) TGGGAAGCCGAACATACTGAA, (reverse) TGCTTAACTCTGCAGGCGTATGTA. The real-time PCR analysis was performed on ABI Prism 7700 using Sequence Detector System version 1.7a (ABI Prism). Relative expression levels of *Cxcl5* were calculated as the number of molecules per 1000 molecules of *B2m* (1000/2^Ct *Cxcl5*-Ct *B2m*^).

### Immunofluorescence on Frozen Sections

Following asphyxiation via CO_2_ administration, eyes were immediately removed and immersion fixed in 4% paraformaldehyde (PFA) for 2 hours at 4°C, then cryoprotected at 4°C in a graded series of sucrose. Eyes were embedded in optimal temperature cutting compound (OCT) and sectioned at 10 µm thickness. Sections were blocked in phosphate buffered saline (PBS) with 0.1% Triton X-100 and 2% normal donkey serum for 20 minutes at room temperature. Sections were then incubated at 4°C overnight in primary antibody solution. The primary antibodies used were myeloperoxidase (1∶200, Novocastra), myeloperoxidase (1∶500, R&D Systems), F4/80 (1∶100, Serotec), CD3 (1∶100, Abcam), CD45R/B220 (1∶50, BD Biosciences), CD31 (1∶50, BD Pharmingen) and mLIX (CXCL5) (1∶200, ImmunoKontact). Sections were rinsed in PBS, and incubated with Alexa Fluor 488 (Invitrogen) or Cy3 (Jackson Immunoresearch) conjugated secondary antibody (1∶400) for 45 minutes at room temperature. Slides were counterstained with 4′,6-Diamidino-2-phenylindole dihydrochloride (DAPI, 1∶200, Sigma). Images were captured on an Eclipse E600 microscope (Nikon; Tokyo, Japan) using a SPOT camera (Spot Diagnostics; Sterling Heights, MI) or a Zeiss 510 Confocal Laser Scanning System and Axio Imager Microscope using LSM 510 Software (release 4.2) (Carl Zeiss MicroImaging, Inc., Thornwood, NY). Immunofluorescence was performed on wild-type controls with matched genetic background for *Dstn^corn1^* and *Dstn^corn1-2J^* mutant mice (A.BY wild type and B6 wild type, respectively). Since no differences in immunofluorescent staining patterns were observed between A.BY and B6 wild-type mice, representative images of A.BY wild type cornea are shown in Figure 1 and 3.

### Histological Quantification of Inflammatory Cells

Corneal frozen sections were stained for the neutrophil marker, myeloperoxidase [21], [22], macrophage marker, F4/80 [23], T cell marker, CD3 [24], and B cell marker CD45R/B220 [25], [26]. Two separate sections were analyzed for each eye. To assess the presence and distribution of cells in the cornea we used a method similar to that described in Hall *et al.* (1999) [62]. Peripheral, paracentral, and central regions of the cornea were defined as distance from the limbus. Cell numbers for each section were determined from limbus to limbus, which include two peripheral regions (0 to 150 µm from each limbus), two paracentral regions (150–300 µm from each limbus), and one central region that was the remainder of the cornea. Cell numbers for the central region were normalized to a total of 300 µm, a length equal to that measured for the peripheral and paracentral regions. Numbers for all regions were combined to yield values representing cell recruitment for the whole cornea. Numbers for each region were combined when showing the distribution of recruited cells. Cells were counted using ImageJ software (http://rsb.info.nih.gov/ij) on digital images taken using the Spot Image Analysis system. At least three eyes were examined per group.

### Flow Cytometry

Blood from A.BY WT and *Dstn^corn1^* mice was collected via tail vein bleeds and immediately suspended in cold 1X PBS solution. Cells were pelleted by centrifugation and briefly resuspended in 5 ml of Red Blood Cell (RBC) Lysis Buffer (.155M NH_4_Cl, .01M KHCO_3_, 4×10^−4^M EDTA in ddH_2_0, pH 7.2–7.4) to lyse the RBCs by hypotonic shock. After a short time, 9 ml of 1X PBS was added to stop further lysis. This step was repeated and remaining cells were pelleted by centrifugation. Cells were resuspended in Flow Staining Buffer (5% Calf Serum, .1% Sodium Azide, 1X PBS) and kept on ice until analyzed. Samples were analyzed on a FACScalibur™ using CELLQuest™ software (BD Biosciences, Franklin Lakes, NJ). Cells were sorted according to size (FSC-H, Forward Scatter) and granularity (SSC-H, Side Scatter), which facilitates the distinct identification of granulocyte, lymphocyte, and monocyte populations in the sample [63]. Data were gated and relative percentages of each leukocyte type were determined using FlowJo Software (Version 8.7). Three animals were examined per genotype, and at least 20,000 events were sorted per animal.

### Peripheral Blood Smear Quantification

Blood from A.BY WT and *Dstn^corn1^* mice was collected via tail vein bleeds and smeared onto slides. Blood was dried completely and slides were then stained with Diff-Quick (Dade Behring Inc., Newark, DE) according to the manufacturer's protocol. Briefly, slides were immersed five times in fixative solution (1.8 mg/liter Triarylmethane Dye in methyl alcohol), five times in Solution I (1 g/liter Xanthene Dye, buffer, and .01% sodium azide), five times in Solution II (0.625 g/liter Azure A and 0.625 g/L Methylene Blue), and thoroughly rinsed in deonized water. Each immersion was for one second. Slides were mounted and viewed under 400X magnification. For each animal, 400 nucleated cells were analyzed. Three animals were examined per genotype.

### Immunofluorescence on Whole Mount Cornea

Following asphyxiation via CO_2_ administration, eyes were immediately removed, placed in PBS, and the corneas were isolated. Corneas were fixed overnight in 4% PFA at 4°C. The next day, corneas were rinsed twice in PBS for 10 minutes, post-fixed in 100% acetone for 20 minutes, and rinsed twice in PBS for 10 minutes. Corneas were blocked overnight in PBS with 0.8% Triton X-100 and 2% normal donkey serum, transferred to block solution with CD31 antibody (1∶50; BD Pharmingen) and incubated overnight. Corneas were then transferred to block solution with an Alexa Fluor 488 conjugated secondary antibody (1∶400; Invitrogen) and incubated overnight. The following day, corneas were rinsed in PBS with 2% normal donkey serum six times at 30 minutes each and mounted. All steps following fixation in 4% PFA were performed at room temperature. Representative images were taken on a Radiance 2100 rainbow confocal laser scanning system on a Nikon TE2000 inverted microscope with Lasersharp 2000 software.

### Quantification of the Vascularized Area of the Cornea

Digital images of all corneal flat mounts were collected using the Spot Image Analysis System. Vascularized area and total corneal area were measured using ImageJ software. The total corneal area was outlined using the innermost vessel of the limbal arcade as the border, and the area of CD31-positive vessels within the cornea was then calculated and normalized to the total corneal area (expressed as the percentage of vascularized cornea). At least four corneas were examined per group.

### Statistical Analysis

A *t*-test was performed for statistical comparison of the numbers inflammatory cells in the cornea, the percentages of leukocytes from the flow cytometric and peripheral blood smear analyses, and the relative expression levels of *Cxcl5* between control and mutant mice. A Mann-Whitney test was performed for the quantifications of the vascularized area of the cornea. GraphPad Prism software (GraphPad software, Inc., San Diego, CA) was used for statistical analysis and to create all graphs reporting numerical values. * p<0.05, ** p<0.01, *** p<0.001.
